# Molecular Mechanism for Conformational Dynamics of Ras·GTP Elucidated from In-Situ Structural Transition in Crystal

**DOI:** 10.1038/srep25931

**Published:** 2016-05-16

**Authors:** Shigeyuki Matsumoto, Nao Miyano, Seiki Baba, Jingling Liao, Takashi Kawamura, Chiemi Tsuda, Azusa Takeda, Masaki Yamamoto, Takashi Kumasaka, Tohru Kataoka, Fumi Shima

**Affiliations:** 1 Division of Molecular Biology, Department of Biochemistry and Molecular Biology, Kobe University Graduate School of Medicine, 7-5-1 Kusunoki-cho, Chuo-ku, Kobe 650-0017, Japan; 2 Japan Synchrotron Radiation Research Institute (JASRI), 1-1-1 Kouto, Sayo-cho, Sayo-gun, Hyogo 679-5198, Japan; 3 RIKEN SPring-8 Center, 1-1-1 Kouto, Sayo-cho, Sayo-gun, Hyogo 679-5148, Japan

## Abstract

Ras•GTP adopts two interconverting conformational states, state 1 and state 2, corresponding to inactive and active forms, respectively. However, analysis of the mechanism for state transition was hampered by the lack of the structural information on wild-type Ras state 1 despite its fundamental nature conserved in the Ras superfamily. Here we solve two new crystal structures of wild-type H-Ras, corresponding to state 1 and state 2. The state 2 structure seems to represent an intermediate of state transition and, intriguingly, the state 1 crystal is successfully derived from this state 2 crystal by regulating the surrounding humidity. Structural comparison enables us to infer the molecular mechanism for state transition, during which a wide range of hydrogen-bonding networks across Switch I, Switch II and the α3-helix interdependently undergo gross rearrangements, where fluctuation of Tyr32, translocation of Gln61, loss of the functional water molecules and positional shift of GTP play major roles. The NMR-based hydrogen/deuterium exchange experiments also support this transition mechanism. Moreover, the unveiled structural features together with the results of the biochemical study provide a new insight into the physiological role of state 1 as a stable pool of Ras•GTP in the GDP/GTP cycle of Ras.

The small GTPases Ras, the products of the *ras* proto-oncogenes, consist of three isoforms, H-Ras, K-Ras and N-Ras, in mammals and are frequently activated by amino acid substitution mutations such as G12V and Q61L in a wide variety of human cancers, making them some of the most promising targets for anti-cancer drug development^1^. Ras function as guanine nucleotide-dependent molecular switches regulating cell growth, development and apoptosis by cycling between GTP-bound active and GDP-bound inactive forms (Ras·GTP and Ras·GDP, respectively). Ras·GTP binds directly with the effectors such as Raf kinases through its flexible binding interface consisting of Switch I (residues 32–38) and Switch II (residues 60–75), leading to activation of the downstream signal^2^,^3^,^4^,^5^. The GDP/GTP cycle of Ras is controlled by guanine nucleotide exchange factors (GEFs) and GTPase-activating proteins (GAPs)^5^. GEFs catalyze nucleotide release from Ras·GDP thereby forming a nucleotide-free form, the majority of which is subsequently converted to Ras·GTP because the cellular concentration of GTP is much higher than that of GDP. On the other hand, GAPs accelerate intrinsic GTP-hydrolyzing activity of Ras thereby inactivating Ras.

^31^P NMR analyses revealed that in solution H-Ras in complex with a non-hydrolyzable GTP analogue, guanosine 5′-(β, γ-imido) triphosphate (GppNHp), exists in dynamic equilibrium between at least two distinct conformational states, called state 1 and state 2, which are characterized by different chemical shift values of the nucleotide phosphorus atoms, especially that of the γ-phosphate group^6^. This conformational dynamics represents a common structural feature shared by other members of the Ras-family small GTPases including Rap, Ral and M-Ras although the state distribution exhibits a great variation among them^7^. Because association with the effector molecules such as Raf induced a shift of the equilibrium toward state 2, it was thought that state 2 represents an active form capable of interacting with the effectors while state 1 represents an inactive form with a drastically impaired affinity to the effectors^6^,^8^,^9^. While tertiary structures of state 2 had been solved with wild-type H-Ras (H-RasWT)^10^,^11^,^12^,^13^, those of state 1 were solved with M-RasWT, its P40D mutant and the T35S, G60A and Y32F mutants of H-Ras, all of which predominantly adopt state 1 in solutio^10^,^14^,^15^,^16^,^17^. In addition, the studies using cross seeding method employing H-RasT35S state 1 crystals enabled the determination of a state 1 structure of H-RasWT although the solved structure was compromised by the heavy intermolecular interactions in crystal^18^. These structures revealed a common property of state 1 distinct from state 2, structural dynamism capable of adopting a large ensemble of conformations particularly in the switch regions, which is caused by the loss of the interactions between Thr35 in Switch I and the γ-phosphate of GppNHp. However, limitation of the information on the tertiary structure of H-RasWT in state 1 has precluded the elucidation of the molecular mechanism for the state transition and the physiological function of state 1.

State 1 is also characterized by the possession of surface pockets suitable for drug binding in contrast to state 2^17^, which has been regarded “undruggable” because of the absence of such a pocket^13^. We have been proposing that compounds which fit into these pockets might function as Ras inhibitors by holding Ras in the state 1 “inactive” conformation. This strategy targeting Ras·GTP to block its interaction with downstream effectors seems more promising than the strategy targeting Ras·GDP to block its interaction with GEFs, recently adopted by other researchers^19^,^20^,^21^,^22^, because constitutively activated Ras mutants are likely to escape from the regulation by GEFs owing to the great reduction in their GTPase activity. Such a class of Ras inhibitors, designated as allosteric Ras inhibitors, could be discovered by rational drug design based on the state 1 pocket structures from RasWT or their G12V and Q61L mutants.

In this paper, we succeed in solving the state 1 crystal structure of H-RasWT·GppNHp by applying the recently developed Humid Air and Glue-coating (HAG) method^23^ to crystals representing a state 2 structure with a structural property of an intermediate of the state transition. This is the first to show the effectiveness of the HAG method in investigation of the protein conformational dynamics. The structural analyses combined with NMR-based hydrogen/deuterium (H/D) exchange experiments enable us to clarify a sequence of key molecular events occurred during the state transition. Moreover, the structural feature of H-Ras in state 1, together with the results of the kinetic analysis of GTP dissociation/association, provides an insight into the physiological role of state 1 in the GDP/GTP cycle of Ras.

## Results

### Determination of a State 1 Crystal Structure of H-RasWT by Application of the HAG Method to a State 2 Crystal

**Figure 1:**
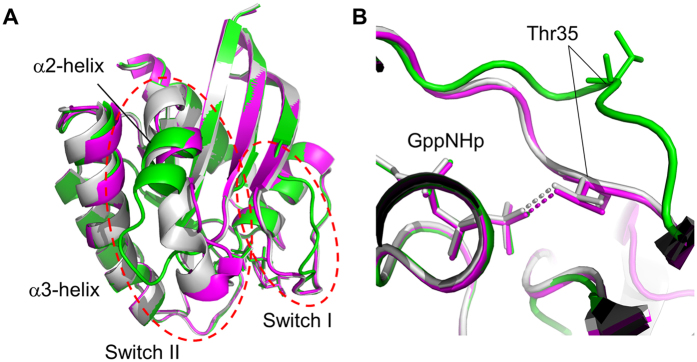
Comparison of the Crystal Structures of State 1^WT^ and State 2* with That of State 2. **(A)** The overall structures of State 2 (gray), State 2* (magenta) and State 1^WT^ (green). The backbone structures were superimposed using the Cα atoms of the residues 1–31, 39–59 and 76–166. Switch I and Switch II are indicated by red dashed lines. (**B**) A close-up view of the direct hydrogen-bonding interactions between Thr35 and the γ-phosphate of GppNHp, represented by dashed lines. GppNHp and the side chains of Thr35 are shown in the stick model.

We solved a new crystal structure of H-RasWT·GppNHp (Fig. 1A) at a resolution of 1.56 Å by using a crystal with a space group *R*32 (Table 1), which was grown under a condition distinct from those reported previously (see Methods). This structure corresponded to state 2 as judged from the presence of the hydrogen-bonding interactions between Thr35 in Switch I and the γ-phosphate of GppNHp (Fig. 1B). Comparison to the known state 2 structures with the same space group under the consideration for minimizing the effects of the crystal packing revealed that the overall backbone structure of the solved state 2 superimposed very well with those of the two reported structures with PDB ID codes 3K8Y and 3V4F^10^,^24^. In particular, it showed extremely high similarity to that of the 3V4F structure (global RMSD value for Cα atoms: 0.125 Å) while showing some differences from the 3K8Y structure; the N-terminal half (residues 62–67) of the α2-helix in Switch II was partially unstructured and the C-terminal part of the neighboring α3-helix was slightly shifted toward the α2-helix (Fig. 1A). Hereafter we call the solved state 2 structure and the 3K8Y structure as “State 2*” and “State 2”, respectively.

Application of the HAG method^23^ to the State 2* crystal induced a sudden lattice transformation along the *a* and *c* axes from 87.97 Å to 91.79 Å and from 132.97 Å to 121.42 Å, respectively, and caused a subtle reduction in the crystal cell unit volume from 8.91 × 10^5^ Å^3^ to 8.86 × 10^5^ Å^3^ (Fig. S1). Using this crystal, we were able to solve another crystal structure with a space group *R*32 at a resolution of 1.60 Å (Fig. 1A and Table 1), which, to our surprise, corresponded to state 1 as judged from the absence of the hydrogen-bonding interactions between Thr35 and the γ-phosphate of GppNHp (Fig. 1B), indicating that the state 2 to state 1 transition had occurred in the crystal. This structure, called “State 1^WT^” hereafter, was suggested to be more suitable for investigation of the state transition mechanism of H-RasWT than the known state 1 structures of H-Ras mutants and H-RasWT derived from cross seeding using H-RasT35S, because it was directly generated from the state 2 structure.

**Figure 2:**
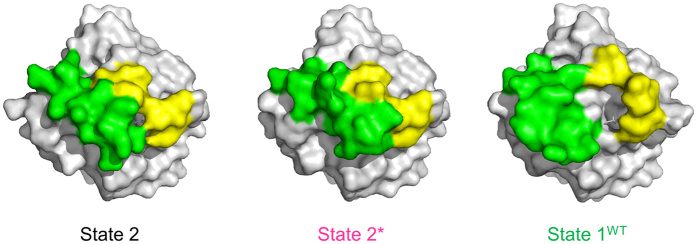
Molecular Surface Characteristics Around the Two Switch Regions in State 2, State 2*, and State 1^WT^. GppNHp is shown in the stick models. Switch I and Switch II are highlighted in yellow and green, respectively.

As observed in the known state 1 structures such as those of M-Ras·GppNHp^17^ and H-RasT35S·GppNHp^15^, the loss of the Thr35-γ-phosphate interaction in State 1^WT^ caused marked deviation of the Switch I loop away from GppNHp, which resulted in the expansion of the accessible surface area from 7.8 × 10^3^ Å^2^ to 8.2 × 10^3^ Å^2^ and the formation of a surface pocket unseen in State 2 and State 2* with the volume of more than 350 Å^3^ surrounded by the two switch regions and the nucleotide (Fig. 2). The pocket structure of State 1^WT^ showed a substantial difference from that observed in the state 1 crystal structure of M-RasP40D·GppNHp used as a target for our previous structure-based drug design (SBDD) of Ras inhibitors^25^ (Fig. S2). In addition, compared to State 2, the N-terminal half of the α2-helix was unstructured forming a loop, which was oriented toward the α3-helix and the C-terminus of the α3-helix was slightly shifted toward the α2-helix (Fig. 1A).

### Role of Tyr32 Fluctuation in Outward Deviation of the Switch I Loop in State 1^WT^

**Figure 3:**
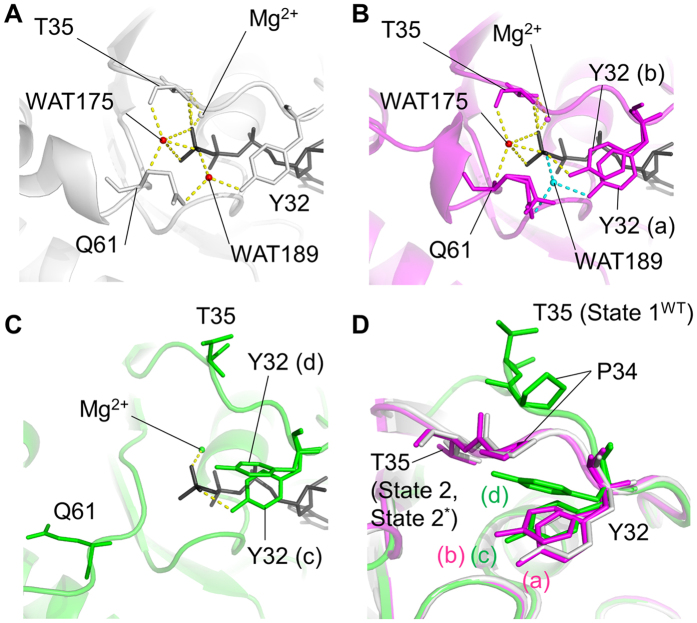
Comparison of the Hydrogen-Bonding Networks Involving Tyr32, Thr35, Gln61 and GppNHp among State 2 (**A**), State 2* (**B**) and State 1^WT^ (**C**). Tyr32, Thr35 and Gln61 and GppNHp (dark grey) are shown in the stick model. Two functional water molecules, WAT175 and WAT189, and hydrogen bonds are represented by red spheres and yellow dashed lines, respectively. WAT189 present in State 2* adopting the conformation (a) and its hydrogen-bonding interactions with the neighboring residues are shown by a sphere and dashed lines colored in cyan. (**D**) Superimposition of the Switch I residues in State 2, State 2* and State 1^WT^. State 2, State 2* and State 1^WT^ are superimposed as described in Fig. 1. Tyr32, Pro34 and Thr35 and GppNHp are shown in the stick model.

**Figure 4:**
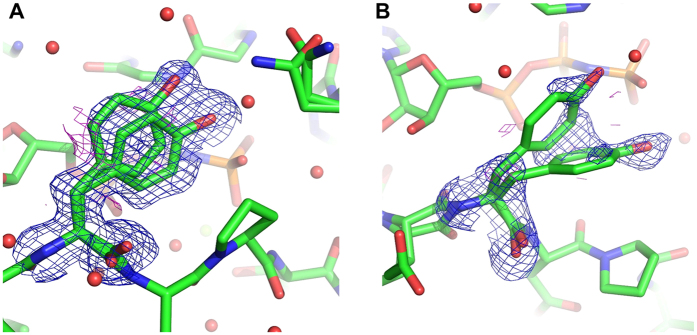
Fo-Fc omit electron density maps for Tyr32. Fo-Fc omit electron densities are shown at 2.0 σ (blue) and −2.0 σ (magenta) levels in State 2* (**A**) and State 1 ^WT^ (**B**).

In State 2 and State 2*, the Switch I loop was mainly fixed by the interactions of Thr35 with GTP, which were mediated by direct hydrogen bonds of its main and side chains and a WAT175-mediated hydrogen bond and a Mg^2+^-coordinated interaction of its side chain with the γ-phosphate of GppNHp (Fig. 3A,B). In contrast, State 1^WT^ lost these interactions altogether, leading to outward deviation of the Switch I loop away from the nucleotide (Fig. 3C). To clarify the molecular mechanism underlying such a drastic conformational change, we first focused on the structures of the Switch I residues. In State 2* and State 1^WT^, Tyr32 was found to exhibit alternative conformations presumably attributed to its flexibility. In State 2*, Tyr32 adopted two distinct conformations, (a) and (b), as shown in Figs 3B,D and 4A. The side chain of the conformation (a) formed a water-mediated interaction with the γ-phosphate of GppNHp as observed with Tyr32 in State 2 while that of the conformation (b) formed a direct hydrogen bond with the γ-phosphate. In State 1^WT^, the side chain of Tyr32 showed lower electron density than that in State 2* indicating much broader fluctuation in the local structure, which includes two alternative conformations, (c) and (d), as the representatives. (Figures 3C,D and 4B). The side chain of the conformation (c) formed a direct hydrogen bond with the γ-phosphate as observed with the conformation (b) in State 2*. On the other hands, the conformation (d) exhibited a marked deviation toward the adjacent Pro34 and were located at the positions capable of causing a steric hindrance to the Pro34 side chains in State 2 and State 2* (Fig. 3D). This implied that the conformational change of Tyr32 would contribute to the outward movement of the Switch I loop during the state 2 to state 1 transition by dislocating Pro34 and thereby causing an outward positional change of Thr35.

### Structural Changes in Switch I Accompanied by the Loss of the Functional Water Molecules

Based on the previous studies on the state 2 structures of H-RasWT·GTP alone or in complex with GAP, it was widely recognized that a nucleophilic attack by a precatalytic water molecule WAT175 (Fig. 3A) on the γ-phosphate represents a key step for hydrolysis of GTP^10^,^13^,^26^,^27^. Further, recent studies showed that another water molecule WAT189, which bridges the γ-phosphate oxygen atom to both the hydroxyl group of Tyr32 and the carbonyl oxygen atom of the Gln61 side chain (Fig. 3A), also plays a critical role for the GTP hydrolysis by stabilizing the functional residues surrounding GTP and thereby allowing them to assume a conformation suitable for the hydrolysis^10^,^11^.

In State 1^WT^, WAT175 which mediates hydrogen-bonding interactions with Thr35, Gln61 and the γ-phosphate of GppNHp in State 2, was totally missing (Fig. 3C). Meanwhile, we could observe a water molecule at the position corresponding to WAT189 and this water molecule failed to make any interactions with Tyr32 and Gln61 unlike the case with WAT189 in State 2. Thus, it was a free and nonfunctional water molecule incorporated into State 1^WT^. In contrast, State 2* possessed WAT175, which established a full bunch of the interactions with Thr35, Gln61 and the γ-phosphate as observed in State 2 (Fig. 3B). On the other hand, WAT189 retaining its functional interactions with Tyr32, Gln61 and the γ-phosphate in a manner similar to that in State 2 was observed only for State 2* adopting the conformation (a) (Fig. 3B). In the other alternative conformation adopting the conformation (b), hereafter representing State 2* in this paper, WAT189 was missing and the Tyr32 side chain showed a shift in its orientation and established a direct hydrogen bond with the γ-phosphate oxygen atom (Fig. 3B). As already mentioned, the conformation (b) matched the conformation (c) of State 1^WT^. These structural characteristics suggested that the State 2* was an intermediate of the state transition because it shared the loss of WAT189 and the accompanying conformational change of the Tyr32 side chain with State 1^WT^ while retaining the interactions between Thr35 and the γ-phosphate, the criterion for state 2 (Fig. 3B,D).

### Gross Positional Change of the Gln61 Side Chain Assisted by the Residues in the α2- and α3-Helices During the State Transition

**Figure 5:**
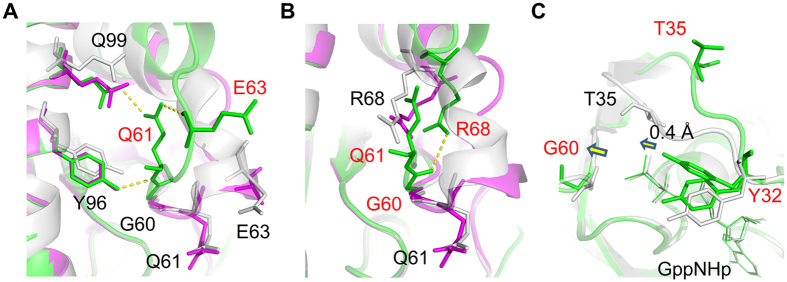
Comparison of the Hydrogen-Bonding Networks Involving the Switch II, α3-Helix Residues and the γ-phosphate of GppNHp. State 2, State 2* and State 1^WT^ are superimposed and colored as described in Fig. 1. Residues of State 1^WT^ shown by red characters exhibited marked positional changes from those in State 2 and State 2*. Yellow dashed lines represent hydrogen bonds. (**A**) Hydrogen-bonding networks involving Gln61 in State 1^WT^. (**B**) Hydrogen bond between Gly60 and Arg68 in State 1^WT^. (**C**) Positional shifts of Gly60 and the γ-phosphate of GppNHp accompanying the state transition from State 2 to State 1^WT^. The positional shifts of the main chain of Gly60 and the γ-phosphate of GppNHp are shown by yellow arrows. GppNHp is shown by the line model.

**Figure 6:**
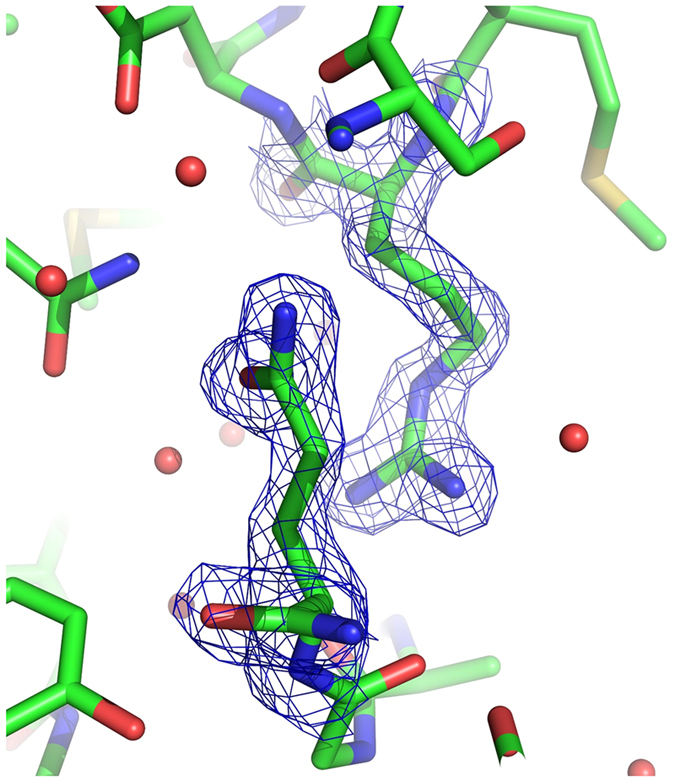
Electron density (2F_O_ − *m*F_C_) maps. 2Fo-Fc electron density in State 1^WT^ is shown at 1.0 σ level around Gln61 and Arg68.

In State 1^WT^, the side chain amide group of Gln61, which plays an essential role in a nucleophilic attack on the γ-phosphate by WAT175 for the GTP hydrolysis, showed a drastic displacement orienting toward an opposite direction compared to those in State 2 and State 2*, which was accompanied by the loss of WAT175 and WAT189 mediating its interactions with Thr35 and Tyr32, respectively, as well as with the γ-phosphate (Figs 3A–C, 5A,B and 6). This orientation of the Gln61 side chain was stabilized by direct hydrogen bonds of its amide group with the main chain of Glu63 in the α2-helix and the side chain of Gln99 in the α3-helix as well as of its main chain amide with the side chain of Tyr96 in the α3-helix (Fig. 5A). In State 2*, the side chain of Gln61 seemed to acquire more conformational flexibility due to the loss of the WAT189-mediated hydrogen bond with the side chain of Tyr32 compared to that in State 2 although its position was not so much different (Fig. 3B and 5A,B). Moreover, the positions of the Tyr96 and Gln99 side chains in State 2* were almost indistinguishable from those in State 1^WT^ and appeared ready to accept and stabilize the Gln61 side chain at its position in State 1^WT^ when the conformational change of the Gln61 side chain has occurred during the state 2 to state 1 transition (Fig. 5A). These findings further supported our hypothesis that State 2* might represent an intermediate of the state transition.

Since the side chain of Gln61 in State 1^WT^ was located in the space occupied by those of Arg68 in State 2 and State 2*, its drastic conformational change accompanying the state 2 to state 1 transition was likely to cause dislocation of the Arg68 side chain to a position closer to Gly60, allowing an establishment of a direct hydrogen bond in State 1^WT^ (Figs 5B and 6). The formation of the Gly60-Arg68 hydrogen bond in State 1^WT^ presumably induced a positional change of Gly60, which seemed to account, at least in part, for a slight positional shift of its hydrogen-bonding partner, the γ-phosphate of GppNHp, with the distance of approximately 0.4 Å orienting toward Gly60 but away from the side chains of Tyr32 and Thr35 (Fig. 5C).

**Figure 7:**
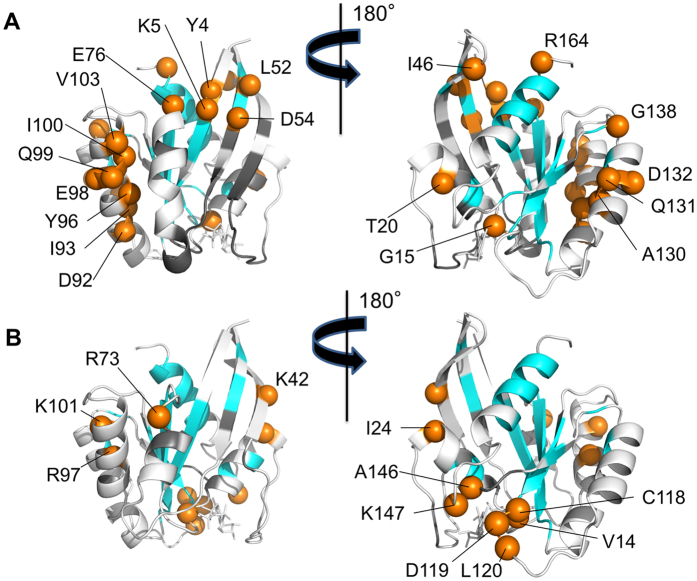
Distinct Dynamic Properties of Various Residues in H-RasWT·GppNHp and H-RasT35S·GppNHp Revealed by the Hydrogen/Deuterium (H/D) Exchange Experiments. Shown by orange spheres are residues of H-RasWT·GppNHp showing less solvent-protection compared to the corresponding residues of H-RasT35S·GppNHp by a factor of lower than 0.8, *i.e*. PF_H-RasWT_/PF_H-RasT35S_ < 0.8, (**A**) and residues of H-RasT35S·GppNHp showing less solvent-protection compared to the corresponding residues of H-RasWT·GppNHp by a factor of lower than 0.8, *i.e*. PF_H-RasT35S_/PF_H-RasWT_ < 0.8 (**B**), which are depicted in State 2 and State 1^WT^, respectively. See Table S1 for the PF values and the apparent hydrogen-deuterium exchange rate (*k*_ex_) of the individual residues. Structured regions and residues whose PF values could not be obtained because of signal overlapping and broadening are shown in cyan and black colors, respectively.

Our studies described above suggested a crucial role of the rearrangements of the hydrogen bonding networks mediated by the α3-helix residues facing the α2-helix during the state transition in H-RasWT. To further investigate the dynamic properties of the α3-helix relevant to the conformational transition, we calculated the residue-specific protection factor (PF), which is an index of protection of a particular amino acid residue from the bulk solvent, based on the NMR-based H/D exchange experiments^28^,^29^. Calculations of the PF values were done with H-RasWT·GppNHp, assuming state 1 and state 2 in the 36 ± 2 to 64 ± 2 ratio, and H-RasT35S·GppNHp, almost exclusively assuming state 1, and the values for particular residues of the two proteins were compared with each other. As a result, the α3-helix residues, such as Asp92, Ile93, Tyr96, Glu98 and Val103, showed significantly lower PF values in H-RasWT compared to the corresponding residues in H-RasT35S (Fig. 7A and Table S1). Although the signals for Gln99 and Ile100 were not unambiguously assigned because of overlapping, we could at least conclude that either one or both of the signals showed higher PF values in H-RasT35S because the corresponding signal intensities in H-RasWT were too low to be detected (Fig. S3). Intriguingly, these more highly solvent-exposed α3-helix residues in H-RasWT had spatially adjacent location facing the α2-helix (Fig. 7A). Likewise, O’Connor *et al*. had shown the existence of the conformational dynamics in the α3-helix of H-RasWT·GppNHp by ^15^N spin relaxation rcCPMG measurements^30^. These results agreed well with our crystallographic observations that the α3-helix residues facing the α2-helix underwent conformational rearrangements in association with the state transition.

### Higher GTP Association Rate of State 1 Compared to State 2

The results of the NMR-based H/D exchange experiments also revealed that residues located in the guanine base-binding motifs NKCD (residues 116–119) and SAK (residues 145–147), such as Cys118, Asp119, Ala146 and Lys147, exhibited lower PF values in H-RasT35S·GppNHp compared to those in H-RasWT·GppNHp (Fig. 7B and Table S1), indicating that the corresponding regions were more exposed to the solvent in the state 1 conformation. This also implied that GppNHp bound to Ras was more exposed to solvents in the state 1 conformation, which was consistent with a higher degree of solvent exposure of GppNHp in the state 1 conformation due to a marked outward deviation of switch I and switch II, which covered GTP in the state 2 conformation, as observed in the known crystal and NMR structures of state 1 including State 1^WT^. We had previously shown that Ras-family small GTPases with higher populations of state 1 exhibit higher dissociation and association rate constants for GTP^7^. These observations prompted us to measure the intrinsic association and dissociation rates, *k*_on_ and *k*_off_ values, respectively, of state 1 and state 2 for guanosine 5′-(γ-thio)-triphosphate (GTPγS). To this end, we again used H-RasWT and H-RasT35S by taking advantage of the fact that H-RasT35S exhibited a higher proportion of state 1 compared to H-RasWT even in the GTPγS-bound forms^9^. As a result, H-RasT35S showed the substantially higher *k*_on_ and *k*_off_ values compared to those of H-RasWT while the *K*_d_ values for GTPγS were almost identical with each other (Table S2), indicating that state 1 had the higher *k*_on_ value for GTP compared to state 2.

## Discussion

We were able to solve the state 1 crystal structure of H-RasWT·GppNHp, State 1^WT^, by achieving the conformational transition to state 1 through the application of the HAG method to the crystals representing State 2*, which turned out to be nearly identical with the 3V4F structure^24^. The HAG method was originally developed for the improvement of diffraction data quality by dehydration of the crystals having loose molecular packing and large water content^23^. In the present study, the State 2* crystal was coated with polymer glue consisting of polyvinylalcohol and subjected to the atmosphere with reduced relative humidity (87.95% RH), which presumably dehydrated the crystals and brought about a change in the bulk solvent distribution in them. This change somehow facilitated a conformational transition toward state 1, which was accompanied by a lattice transformation, and eventually transformed the State 2* crystal into the State 1^WT^ crystal. Such a drastic structural change by application of this method has been unprecedented^23^. It seemed unlikely that the State 1^WT^ formation was significantly affected by the changes in the mode of interactions with neighboring H-Ras molecules in the crystal because most of the surface areas interacting with the neighboring molecules in the State 2* crystal did not overlap with the regions undergoing major conformational changes upon state transition, typically Switch I and Switch II (Fig. S4B).

**Figure 8:**
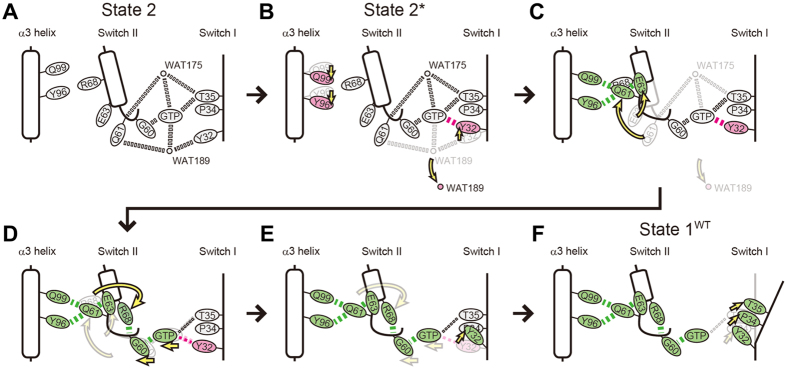
Molecular Mechanism for the State 2 to State 1 Transition Inferred from the State 1^WT^ and State 2* Structures. Residues, WAT175, WAT189 and hydrogen-bonding interactions (dotted lines), whose configurations match those in State 2, are shown in a gray color while those whose configurations exhibit significant changes in State 2* and State 1^WT^ are shown in magenta and green colors, respectively. Conformational changes are represented by yellow arrows.

Comparisons among State 1^WT^, State 2* and State 2 allowed us to infer a series of events occurred during the state transition of H-RasWT·GTP. State 2* seemed to represent an intermediate between State 1^WT^ and State 2 because it shared some key properties not only with State 1^WT^, such as the absence of WAT189, the presence of the Tyr32-γ-phosphate hydrogen bond and the positions of the Tyr96 and Gln99 side chains, but also with State 2, such as the presence of the Thr35-γ-phosphate hydrogen bonds, the hallmark of state 2, and WAT175. Moreover, State 2* adopting the conformation (a) retained the WAT189-mediated hydrogen bonds with Tyr32, Gln61 and the γ-phosphate like State 2. Assuming that serial structural changes occurred in the order of State 2, State 2* and State 1^WT^, we were able to infer the following pathway for the state 2 to state 1 transition depicted in Fig. 8. The conversion of State 2 (Fig. 8A) to State 2* (Fig. 8B) is associated with the loss of WAT189, which mediates interactions among Tyr32, Gln61 and the γ-phosphate of GppNHp in State 2, and the displacement of the Tyr32 side chain, forming a hydrogen bond with the γ-phosphate, represented by the conformation (b) (Fig. 3B). As previously discussed by Buhrman *et al*., this structural conversion could be partly relevant to the allosteric events occurred in the vicinity of the plasma membrane^10^,^31^. During this process, Gln61 is still fixed by the WAT175-mediated interactions with Thr35 and the γ-phosphate. Subsequently, H-Ras·GppNHp undergoes a series of events involving the loss of WAT175 and a drastic translocation of Gln61. Presumably, the loss of WAT175 mobilizes Gln61, which is subsequently trapped by the scaffold formed by Tyr96 and Gln99 located in the side of the α3-helix facing the α2-helix through establishment of hydrogen bonds (Fig. 8C). Also, this is accompanied by a dislocation of Glu63 in switch II and formation of a hydrogen bond with Gln61 (Fig. 8C). The grossly altered conformation of the Gln61 side chain induces dislocation of the Arg68 side chain to a position closer to Gly60, resulting in formation of a hydrogen bond between them (Fig. 8D). These events collectively induce a deviation of the switch II loop away from GppNHp as well as a slight positional shift of GppNHp orienting toward Gly60 but away from the side chains of Tyr32 and Thr35 through the positional change of Gly60 and Gln61. A similar positional change of GppNHp was observed upon the state 2 to state 1 transition of M-Ras·GppNHp^15^. This positional shift of GppNHp is expected to weaken the interactions of its γ-phosphate with Tyr32 and Thr35. This presumably induces the further positional shift of Tyr32 represented by the conformation (d) (Fig. 3C), which loses the hydrogen bond with the γ-phosphate and exhibit a marked deviation toward Pro34 (Fig. 8E). This results in dislocation of Pro34 and the outward movement of the switch I loop, finally leading to the breakage of the Thr35-γ-phosphate hydrogen bonds and a further outward deviation of the switch I loop in State 1^WT^ (Fig. 8F).

Thus, the hydrogen-bonding interaction of the Tyr32 side chain with the γ-phosphate, whether direct or mediated by WAT189, seems to function as an anchor for Tyr32 to avoid its collision with the Pro34 side chain, thereby stabilizing state 2. In other words, the positional shift of Tyr32, induced by formation of a direct hydrogen bond with the γ-phosphate, seems to be necessary for the establishment of the Thr35-γ-phosphate hydrogen bond during the state 1 to state 2 transition. This is consistent with the observation that introduction of Y32F substitution into H-Ras caused a significant increase in the state 1 population^32^. Although our present results provided little information on the molecular mechanism for the opposite path, *i.e*. state 1 to state 2 transition, we can envisage that incorporation of the two functional water molecules, WAT175 and WAT189, in their proper configurations would be essential for this process by mediating interactions among Tyr32, Thr35, Gln61, Mg^2+^ and GTP and facilitating the hydrogen bonding interactions of Thr35 and Gly60 with the γ-phosphate. Future studies using an advanced HAG method capable of more gradual humidity control may enable us to solve additional crystal structures with state intermediate characteristics and further dissect the molecular mechanisms underlying the state dynamics. It must be noted that various hydrogen-bonding interactions relevant to the state transition are known to exhibit strong interdependence, often mediated by guanine nucleotide, as evidenced by marked rearrangements of the hydrogen-bonding networks caused by single amino acid substitutions in H-Ras, resulting in drastic alteration of the state equilibrium^6^,^14^,^16^,^32^. Considering such a complex nature of the structural rearrangements, it is possible that multiple pathways might exist in parallel for the state conversion, the selection of which might be influenced by the molecular environments.

The interconverting nature of Ras·GTP between the active state 2 and the inactive state 1 led to the concept that state 1 stabilizers might function as allosteric Ras inhibitors. Kalbitzer and colleagues reported that metal cyclens bind to and stabilize state 1 of H-Ras^33^,^34^,^35^,^36^. However, these compounds inhibited Ras-Raf binding *in vitro* only weakly with the *K*_d_ value higher than 10^−3^ M and their cellular activity remained untested. In this study, a state 1-specific surface pocket located apart from the metal cyclen-binding site was discovered on H-RasWT·GppNHp, whose size and shape were quite different from that of M-RasP40D·GppNHp used as a target for our previous SBDD study (Fig. 2 and Fig. S2). Application of the HAG method to the structural determination of the surface pockets of the oncogenic Ras mutants such as H-RasG12V and H-RasQ61L, followed by the SBDD, may lead to the discovery of allosteric Ras inhibitors with a potency suitable for clinical application.

So far, little is known about the physiological function of state 1. It is known that H-Ras mutants predominantly adopting state 1, such as those carrying Y32F, T35S and G60A mutations, had their intrinsic GTPase activity drastically impaired and that stabilization of state 2 by effector binding, on the contrary, increased the GTPase activity^8^. In this study, we showed that State 1^WT^ actually lost the two water molecules essential for the intrinsic GTP hydrolysis, WAT175 and WAT189, providing a structural basis for the reduced GTPase activity of state 1. Moreover, we showed that H-RasT35S exhibited the substantially higher association rate for GTP compared to that of H-RasWT (Table S2). This result, together with the result of the NMR-based H/D exchange experiment showing that the guanine base-binding residues are more exposed to the solvent in state 1 (Fig. 7B and Table S1), indicated that state 1 represents a more favorable conformation for GTP incorporation than state 2. Although state 1 also exhibited a substantial increase in the dissociation rate for GTP, it is expected to be converted to state 2 before GTP dissociation takes place. This is because the GTP dissociation rate is in the order of 10^−4^ sec^−1^, which is much slower than the rate for state transition from state 1 to state 2 with the order of 10^0^ sec^−1^ 8. Based on these findings, we propose the following model for the GDP/GTP cycle of Ras (Fig. S5). The nucleotide-free Ras, generated by the action of GEFs on Ras·GDP, incorporates GTP and preferentially adopts state 1. Owing to its lower GTPase activity, state 1 is capable of forming a more stable pool of Ras·GTP than state 2. Subsequently, state 1 is converted to state 2, the active form, in response to an equilibrium shift toward state 2 induced by the reduction of the free state 2 concentration, resulting from effector binding or intrinsic GTP hydrolysis.

The present study shows a potential of general application of the HAG method for structural biology studies on the conformational dynamics of proteins. In solution, proteins intrinsically fluctuate among multiple conformations, which undergo dynamic equilibrium. Investigation of their structural properties including minor-populated components through experimental and computational approaches, including CPMG NMR, room temperature X-ray crystallography and high pressure NMR, will help the complete understanding of the molecular basis of the protein functions, such as molecular recognition, enzymatic activity and allosteric regulation^37^,^38^,^39^,^40^,^41^. In this context, the HAG method may become one of the powerful approaches to directly obtain the structural information on each component undergoing an equilibrium at the atomic level.

## Methods

### Protein Purification

Residues 1-166 of H-RasWT and H-RasT35S were expressed as fusions with glutathione *S*-transferase in *Escherichia coli* BL21 (DE3) by using pGEX-6P-1 vector (GE Healthcare, Buckinghamshire, UK), immobilized on glutathione Sepharose 4B resin (GE Healthcare) and eluted by on-column cleavage with Turbo3C protease (Accelagen, California, USA). The H-Ras polypeptides were further purified by chromatography on a HiTrap Q HP column (GE Healthcare), loaded with GppNHp and used for crystallization or NMR spectroscopy experiments and for nucleotide association and dissociation assays as described before^7^.

### Crystallization of H-RasWT·GppNHp and X-Ray Data Collection

Purified human H-RasWT was dissolved in 50 mM Tris·HCl (pH 7.4), 50 mM NaCl, 5 mM MgCl_2_ and 1 mM DTT. The crystal of State 2* was grown by the sitting drop vapor diffusion method at 20 °C with 1 μl of protein solution mixed with 1 μl of a reservoir solution [30% PEG400 (weight/vol), 0.1 M acetate (pH 4.5) and 0.2 M Ca(OAc)_2_] for 7 days. X-ray data for the State 2* crystal were obtained at a wavelength of 1 Å by using the conventional cryocooling technique; the crystal was flash-cooled to 100 K with a nitrogen cryostream. On the other hand, data collection for the State 1^WT^ crystal was performed by the application of the HAG mounting method^23^ as follows. The crystal was picked up and coated with aqueous polymer glue containing 8% (weight/vol) polyvinylalcohol (average molecular weight 4500) and the atmosphere was adjusted to 87.95% relative humidity. After exposure to the humid air for 20 min, the lattice transformation was observed by X-ray diffraction under the ambient temperature. The crystal was flash-cooled with the cryostream and subjected to the diffraction data collection at SPring-8 BL38B1.

### Crystal Structure Determination and Refinement

Diffraction data processing and scaling were performed using the HKL-2000 package^42^. Each crystal structure was refined using the coordinates of an H-Ras state 2 structure (PDB entry 3k8y) as an initial model with REFMAC^43^ and the interactive rebuilding process was performed by using Coot^44^. The overall model quality was assessed with MolProbity^45^. The Ramachandran plot statistics (favored/allowed) for State 2* and State 1 are 96.9%/3.1% and 98.2%/1.8%, respectively, and there are no outliers. Atomic coordinates and structure factors have been deposited in the Protein Data Bank (PDB; http://www.rcsb.org) under accession codes 5B2Z and 5B30. The crystallographic parameters of the structure were listed in Table 1. The programs, PyMOL (DeLano Scientific, LLC) and Molecular Operating Environment (MOE) software package (Chemical Computing Group Inc., Montreal, Canada), were used for the further structural analyses.

### NMR Spectroscopy

Uniformly ^15^N-labeled H-RasWT(1-166) and H-RasT35S(1-166) were purified from *Escherichia coli* cells cultured in M9 minimal media containing [^15^N]NH_4_Cl (1.0 g/L) as the sole nitrogen source, loaded with GppNHp and subjected to NMR measurements in buffer A [25 mM phosphate buffer (pH 6.8), 50 mM NaCl and 10 mM MgCl_2_]. NMR-based hydrogen-deuterium exchange experiments were carried out at 25 °C on a Bruker AVANCE III 600 instrument equipped with shielded gradient triple-resonance probes and analyzed with NMRPipe^46^ and NMRView (One Moon Scientific, Inc., New Jersey, USA).

### NMR-Based H/D Exchange Experiments

0.15 mM solutions of ^15^N-labeled H-RasWT(1-166) and H-RasT35S(1-166) in buffer A were lyophilized and redissolved in the same volume of D_2_O. Subsequently, ^1^H-^15^N band-selective optimized-flip-angle short-transient heteronuclear multiple quantum coherence (SOFAST-HMQC) spectra were acquired at variable time points up to 36 h at 25 °C. The apparent hydrogen-deuterium exchange rates (*k*_ex_) were determined by fitting a single exponential function to the decay of the amide proton signal intensities. The protection factors (PFs) were calculated by dividing the intrinsic amide proton exchange rates by the measured amide proton exchange rates^28^,^29^. The intrinsic amide proton exchange rates were derived from the amide acid sequence using the program SPHERE (http://www.fccc.edu/research/labs/roder/sphere/), based on the methods developed by Englander *et al*.^28^,^29^.

### GTP Association and Dissociation Assays and Determination of Kinetic Parameters

GTP dissociation assays were carried out essentially as described before^7^. Briefly, the GTP dissociation reaction was initiated at 37 °C by adding 200-fold molar excess of GTPγS to 1 μM H-RasT35S(1-166) preloaded with 10 μM [γ-^35^S]GTPγS. Aliquots were subjected to filtration through nitrocellulose membranes (0.22-μM pore size) and the membrane-trapped radioactivity was measured by liquid scintillation counting. The *k*_off_ value was calculated by fitting the dissociation curve to a single exponential function. As for GTP association assays, the nucleotide-free form of H-RasT35S(1-166), prepared as described^7^, was incubated with various concentrations of [γ-^35^S]GTPγS in 50 mM Tris·HCl, pH 8.0, 100 mM NaCl and 5 mM MgCl_2_ at 25 °C for 10 min and subjected to filtration through nitrocellulose membranes (0.22-μM pore size), and the membrane-trapped radioactivity was measured by liquid scintillation counting. The *K*_d_ value for GTPγS was calculated by using the Scatchard plot. The *k*_on_ value was calculated as *k*_off_/*K*_d_.

## Additional Information

**How to cite this article**: Matsumoto, S. *et al*. Molecular Mechanism for Conformational Dynamics of Ras•GTP Elucidated from In-Situ Structural Transition in Crystal. *Sci. Rep*. **6**, 25931; doi: 10.1038/srep25931 (2016).

## Supplementary Material

Supplementary Information

## Figures and Tables

**Table 1:** Data Collection and Refinement Statistics.

PDB ID Code	State 2*	State 1^WT^
	5B2Z	5B30
Data collection		
X-ray source	SPring-8 BL38B1	SPring-8 BL38B1
Detector	ADSC Quantum 315R	ADSC Quantum 315R
Wavelength (Å)	1.000	1.000
Space group	*R*32 (*H*32)	*R*32 (*H*32)
Cell dimensions		
*a*, *b*, *c* (Å)	87.97, 87.97, 132.97	91.79, 91.79, 121.42
α, β, γ (°)	90, 90, 120	90, 90, 120
Resolution (Å)	50.0–1.56 (1.62–1.56)^a^	30.0–1.60 (1.63–1.60)^a^
Wilson B factor (Å^2^)	13.1	19.4
No. observed reflections	311,788	279,449
No. unique reflections	28,415	26,280
*R*_sym_ (%)^b^	6.3 (52.1)	5.9 (82.3)
*I*/σ_*I*_	46.1 (6.5)	49.0 (3.8)
Completeness (%)	100. (100.)	100. (100.)
Redundancy	11.0 (10.9)	10.6 (10.0)
Refinement		
Resolution (Å)	25.4–1.56	28.4–1.60
No. reflections	26,971	24,765
*R*_work_/*R*_free_	0.148/0.178	0.167/0.196
No. atoms/*B*-factors (Å^2^)		
Protein	1,420/16.0	1,425/23.2
Ligand/ion	35/10.5	36/18.4
Water	156/26.2	163/33.7
R.m.s. deviations		
Bond lengths (Å)	0.027	0.027
Bond angles (°)	2.5	2.4
Ramachandran outliers/favoured (%)	0/96.6	0/96.6
Rotamer outliers (%)	2.6	3.2
Clashscore, all atoms	5.3	4.9

^a^Values in parentheses are for highest-resolution shell.

^b^*R*_sym_ = Σ_*hkl*_ Σ_*j*_ |*I*_*hkl,j*_ − *I*_*hkl*_|/Σ_*hkl*_ Σ_*j*_ *I*_*hkl,j*_, where *I*_*hkl*_ is the average of symmetry-related observations of a unique reflection. ^c^ *R*_work_ = Σ_*hkl*_ ||*F*_obs_(*hkl*)|−|*F*_calc_(*hkl*)||/Σ_*hkl*_ |*F*_obs_(*hkl*)|. *R*_free_ = the cross-validation *R* factor for 5% of reflections against which the model was not refined.
