# Supplementary material for: Molecular Mechanism for Conformational Dynamics of Ras·GTP Elucidated from In-Situ Structural Transition in Crystal
Source: Sci Rep. 2016 May 16;6:25931. doi: 10.1038/srep25931 (PMC4867591; doi:10.1038/srep25931)
Supplement: Supplementary Information [file srep25931-s1.pdf]

Molecular Mechanism for Conformational Dynamics of Ras•GTP Elucidated from In-Situ Structural Transition in Crystal

Shigeyuki Matsumoto, Nao Miyano, Seiki Baba, Jingling Liao, Takashi Kawamura, Chiemi Tsuda, Azusa Takeda, Masaki Yamamoto, Takashi Kumasaka, Tohru Kataoka, and Fumi Shima

Table S1. H/D exchange data of backbone amides of H-RasWT•GppNHp and H-RasT35S•GppNHp

Residue	H-RasWT k_{ex} (x 10^{-5} s^{-1})	S. D. k_{ex} (x 10^{-5} s^{-1})	PF (x 10^5)	S. D. PF (x 10^5)	H-RasT35S k_{ex} (x 10^{-5} s^{-1})	S. D. k_{ex} (x 10^{-5} s^{-1})	PF (x 10^5)	S. D. PF (x 10^5)
4	2.6	0.16	0.79	0.048	1.6	0.22	1.5	0.21
5	2.4	0.21	2.3	0.2	2.0	0.2	3.1	0.32
14	0.52	0.13	3.1	0.9	0.95	0.15	1.9	0.3
15	Solvent-exposed [a]				2.1	0.21	3.9	0.39
22	Solvent-exposed [a]				0.52	0.24	7.9	4.2
24	0.58	0.23	1.1	0.46	1.6	0.13	0.43	0.035
42	15	0.97	0.53	0.032	31	5	0.3	0.052
46	0.76	0.19	0.97	0.29	0.65	0.19	1.3	0.4
52	4.2	0.22	0.98	0.052	3.4	0.2	1.3	0.079
54	Solvent-exposed [a]				Solvent-protected [b]			
73	15	1.8			Solvent-exposed [a]			
76	Solvent-exposed [a]				6.3	0.46	0.45	0.033
92	Solvent-exposed [a]				4.0	0.28	0.54	0.039
93	0.88	0.15	0.76	0.13	0.56	0.14	1.3	0.32
96	9.1	0.74	0.51	0.043	7.0	0.39	0.74	0.041
97	9.6	1.4	0.77	0.11	26	2.5	0.32	0.032
98	Solvent-exposed [a]				14	1.2	0.22	0.02
99/100 [c]	Solvent-exposed [a]				Solvent-protected [b]			
101	1.5	0.12	1.9	0.15	2.3	0.26	1.4	0.17
103	Solvent-exposed [a]				20	2.4	0.1	0.012
118	2.6	0.17	9.6	0.58	4.7	0.3	6.1	0.39
119	5.1	0.38	1.6	0.11	9.7	0.86	0.91	0.08
120	6.2	0.35	0.15	0.0085	9.5	0.6	0.11	0.0069
130	10	0.59	0.85	0.049	7.4	0.43	1.3	0.076
131	3.6	0.29	1.8	0.14	2.1	0.18	3.4	0.29
132	5.7	0.36	0.76	0.047	4.0	0.17	1.2	0.052
138	22	0.24	0.52	0.052	18	2.1	0.7	0.086
146	2.4	0.18	4.6	0.36	4.0	0.28	3.1	0.22
147	2.7	0.27	1.9	0.18	5.2	0.43	1.1	0.09
164	Solvent-exposed [a]				14	2.1	0.31	0.048

[a] The reliable k_{ex} and PF values could not be determined due to the fast deuterium exchange.

[b] The decay of the signal intensity was hardly observed during the experiments and so it was difficult to obtain reliable values.

[c] These cross peaks were not unambiguously assigned because of the signal overlapping.

Table S2. Comparison of the kinetic parameters for GTPγS between H-RasWT and H-RasT35S

	k_{off} (x 10^{-4} s^{-1})	k_{on} (x 10^{4} M^{-1} s^{-1})	K_d (x 10^{-8} M)
H-RasWT [a]	1.7	0.42	4.0 ± 0.2
H-RasT35S	4.1	0.93	4.4 ± 0.3

[a] The kinetic parameters for H-RasWT were taken from Liao et al., 2008., which employed the identical assay procedures for GTP association and dissociation used in this study.

SI Figures

Fig. S1

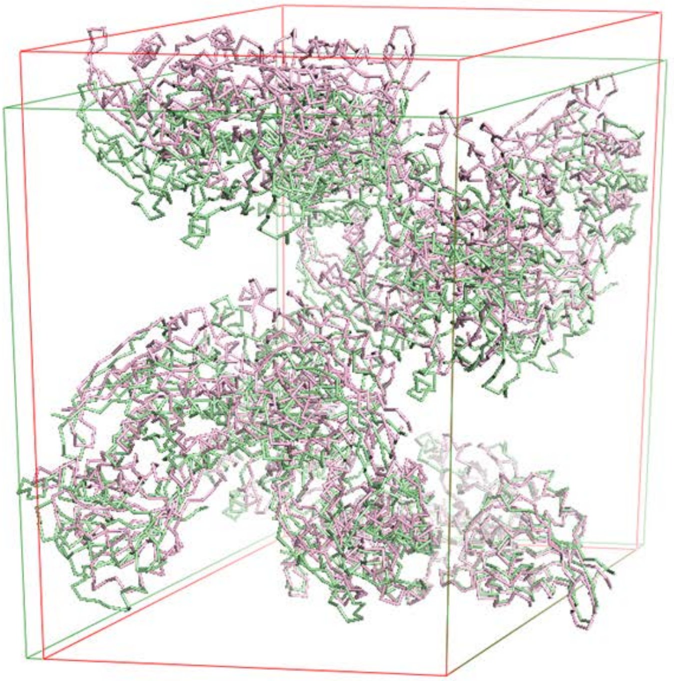

Fig. S2

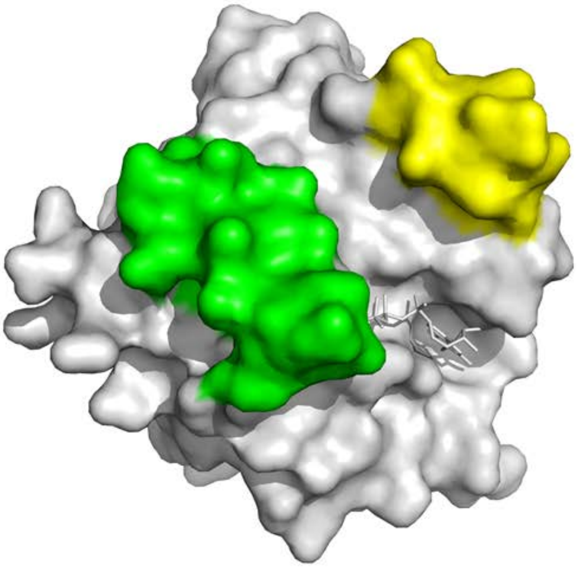

Fig. S3

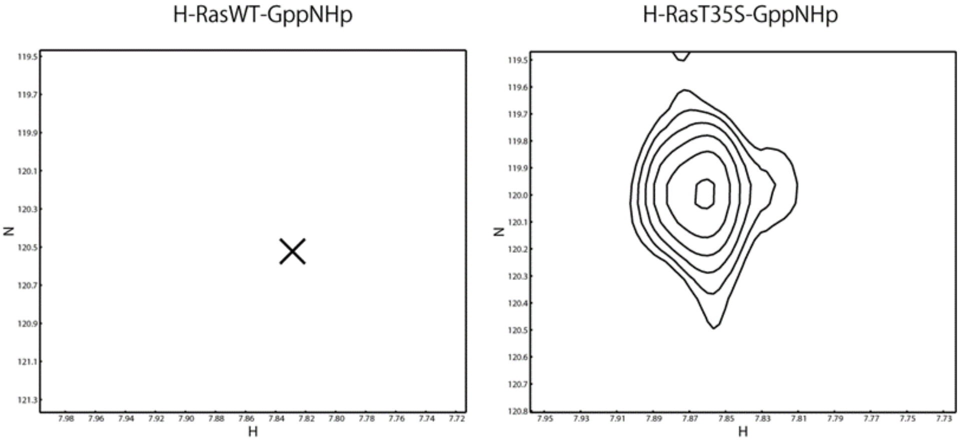

Fig. S4

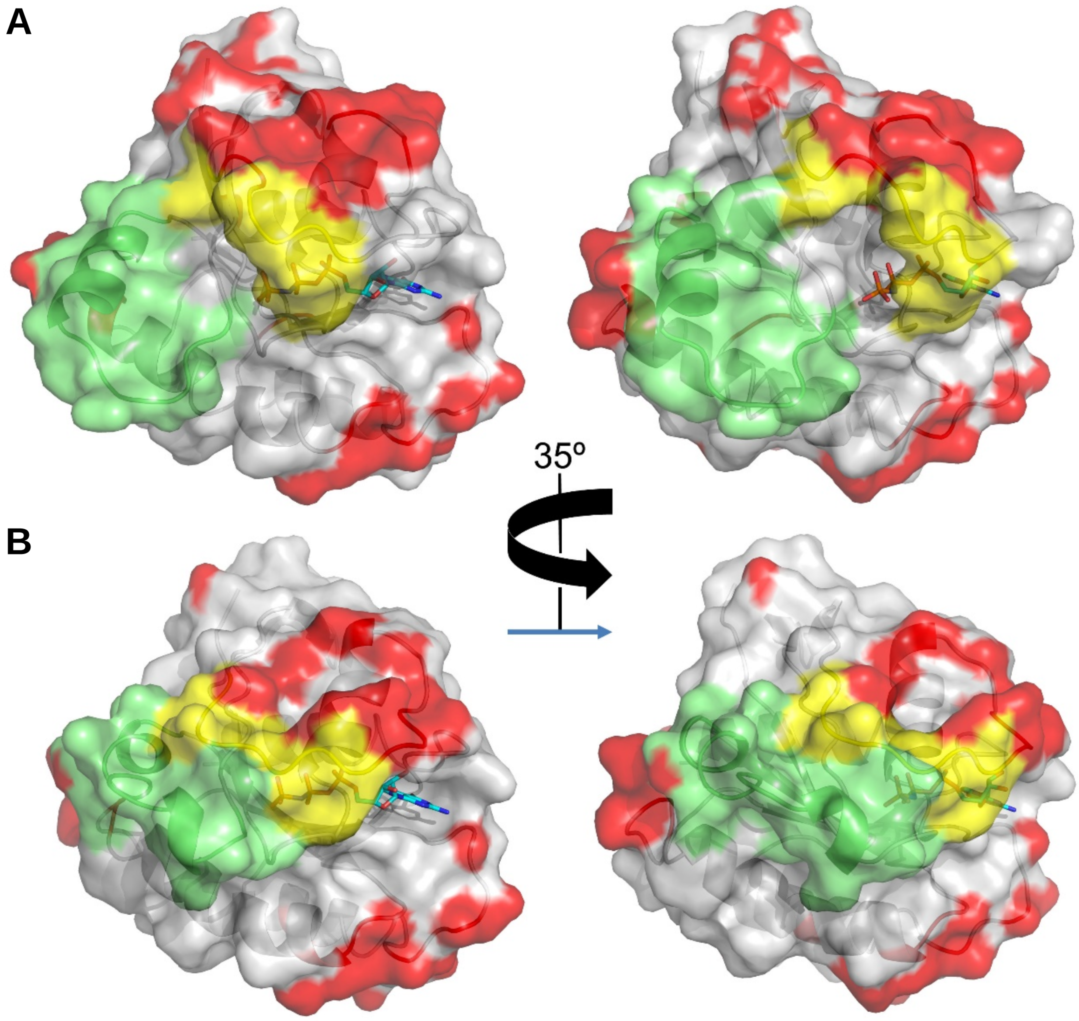

Fig. S5

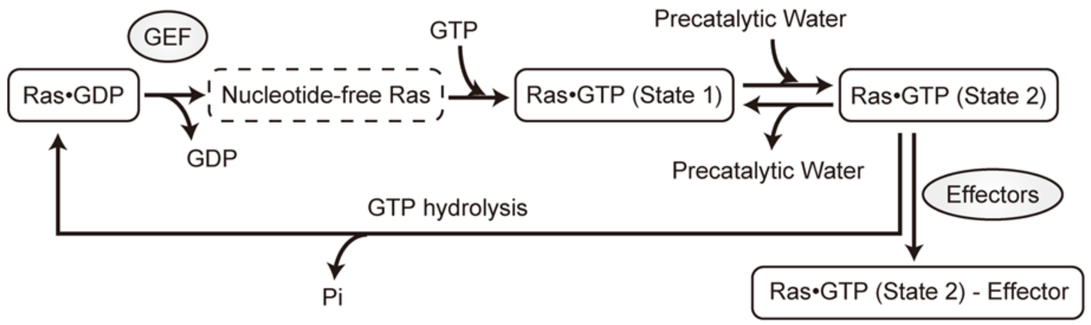
GEF
GTP
Precatalytic Water
Ras•GDP
Nucleotide-free Ras
Ras•GTP (State 1)
Ras•GTP (State 2)
GDP
Precatalytic Water
Effectors
GTP hydrolysis
Pi
Ras•GTP (State 2) - Effector

SI Figure Legends

Fig. S1. Lattice transformation of an H-RasWT•GppNHp crystal induced by the HAG method. The backbone structures in the unit cells and crystal lattices of State 1^{WT} and State 2* are represented by green and magenta lines, respectively. Drastic structural changes in the two switch regions were achieved upon the transition from state 2 to state 1 with a subtle reduction (~0.6%) of the cell volume in a crystal. The major source of the lattice transformation is presumed to be a structural rearrangement at a crystal contact area facing the crystallographic two fold axis as shown in Fig. S4.

Fig. S2. Molecular surface characteristics around the two switch regions in GppNHp-bound M-RasP40D (PDB ID code, 3kkp). GppNHp is shown in the stick models. Switch I and Switch II are highlighted in yellow and green, respectively.

Fig. S3. Amide proton signals of Gln99 and Ile100 in the H/D exchange spectra of H-RasWT•GppNHp and H-RasT35S•GppNHp acquired after 36 hours. The cross peaks of Gln99 and Ile100 were not unambiguously assigned due to overlapping. The cross mark indicates the signal positions of H-RasWT•GppNHp observed in the spectrum without deuterium exchange.

Fig. S4. Crystal packing areas in State 1^{WT} and State 2*. Crystal packing areas in State 1^{WT} (*A*) and State 2* (*B*) are shown by molecular surface representations. Surfaces of the atoms interacting with any atoms in the crystallographic adjacent molecules within the 4 Å distance are colored by red. Switch I and Switch II are highlighted in yellow and green colors,

respectively. GppNHp is shown in the stick model. The direction of the view in the left figures is identical to that in Fig. 1*A*. Most of the residues in Switch I and Switch II, which underwent major conformational changes, were not located on the surface areas interacting with the neighboring molecules in the State 2* crystal. Although only the region closest to the Switch regions consisting of the residues 25-27, 33 and 35-37 was located within 4 Å-distances from the neighboring molecules, no main chain-mediated interaction was observed in State 2*.

Fig. S5. Schematic representation of the GDP/GTP cycle of Ras associated with the state transition. State 1 is preferentially formed from the nucleotide-free Ras generated by the action of GEFs on Ras•GDP. The resulting state 1 without the precatalytic waters acts as a stable pool of Ras•GTP owing to its lower GTPase activity. The functionally active state 2 is recruited from state 1 in response to an equilibrium shift toward state 2 induced by the reduction of the free state 2 concentration, resulting from effector binding or GTP hydrolysis.
